# Prise en charge de la torsion du testicule par un chirurgien généraliste isolé en Afrique

**DOI:** 10.48327/mtsi.v2i2.2022.230

**Published:** 2022-04-04

**Authors:** Thibaut LONG-DEPAQUIT, Paul CHIRON, Stéphane BOURGOUIN, Julie HARDY, François-Xavier DELEDALLE, Julien LAROCHE, Benoit MOLIMARD, Pierre-Henri SAVOIE

**Affiliations:** 1 Service d’urologie, Hôpital d’instruction des armées Sainte-Anne, 2 boulevard Sainte Anne, BP 600, 83000 Toulon, France; 2 Service d’urologie, Hôpital d’instruction des armées Bégin, 69 avenue de Paris, 94160 Saint-Mandé, France; 3 Service de chirurgie viscérale, Hôpital d’instruction des armées Sainte-Anne, 2 boulevard Sainte Anne, BP 600, 83000 Toulon, France

**Keywords:** Torsion testicule, Afrique, Orchidopexie, Orchidectomie, Situation isolée, Testicular torsion, Africa, Orchidopexy, Orchidectomy, Health isolation

## Abstract

L’apparition d’une douleur scrotale brutale et intense pose plusieurs problèmes quand elle survient chez un homme jeune en Afrique. Parmi les étiologies possibles, la torsion testiculaire est l’urgence chirurgicale à écarter, car au-delà de 6 heures d’évolution le pronostic fonctionnel du testicule est engagé. L’évolution septique vers une fonte purulente, en cas de torsion dépassée, est aussi possible. D’incidence légèrement inférieure à celle des pays occidentaux, la méconnaissance de cette pathologie par les acteurs de santé de proximité, la précarité ou l’isolement sanitaire de certaines populations dans certaines régions sous-médicalisées, contribuent aux retards diagnostique et thérapeutique. Cela conduit souvent à une évolution péjorative, la perte du testicule étant directement corrélée au délai de prise en charge. La torsion testiculaire a ainsi été identifiée comme l’une des principales causes d’infertilité masculine en Afrique. Pourtant, le diagnostic clinique et le traitement chirurgical nécessitent peu de moyens et restent accessibles dans un milieu à faibles ressources ou en conditions précaires. Cet article reprend le tableau clinique de torsion du cordon spermatique et la technique d’orchidopexie.

## Abstract (en)

The onset of sudden and intense scrotal pain exposes to several problems when it occurs in a young man in Africa. Among the possible etiologies, testicular torsion is the surgical emergency to rule out, because beyond 6 hours of evolution the functional prognosis of the testicle is concerned. The septic evolution towards a purulent melting, in case of exceeded torsion, is also possible. Of slightly lower incidence than in Western countries, lack of awareness of this pathology by local health actors, the precariousness or health isolation of certain populations in certain under-medicalized regions, contribute to diagnostic and therapeutic delays. This often leads to a pejorative development, loss of the testicle being directly correlated with the delay in treatment. Testicular torsion has thus been identified as one of the main causes of male infertility in Africa. However, clinical diagnosis and surgical treatment require few resources and remain accessible in an environment with low resources or in precarious conditions. Indeed, despite the contribution of ultrasound in certain situations, the clinical picture is at the center of the diagnosis and therapeutic decision making. At the slightest doubt, surgical exploration is necessary. The multi-tunic anatomy of the testicle facilitates its surgical fixation in the event of conservation, ideally by triangulation of single-strand non-absorbable thread. Simultaneous fixation of the contralateral testicle is currently the subject of debate in the literature. In Africa, the benefit/risk balance, taking into account in particular the difficulty of subsequent access to care, justifies, from our point of view, performing contralateral orchidopexy at the same time. Depending on the appearance of the testicle and, to a lesser extent, the duration of the evolution of the symptoms, orchidectomy may be necessary. This article describes the clinical picture of spermatic cord torsion and the orchidopexy technique.

## Introduction

La torsion du cordon spermatique est une urgence fonctionnelle. L’ischémie aiguë du testicule peut mener à la nécrose de la pulpe testiculaire, mettant en jeu son pronostic fonctionnel. Outre le nombre de tours de spire, c’est surtout le délai de prise en charge et la rapidité d’accès au bloc opératoire qui conditionnent la sauvegarde du testicule [14].

Si, dans les pays développés, cette urgence chirurgicale est bien connue du corps médical comme du grand public, entre autres du fait de ses enjeux fonctionnels et médico-légaux [7], c’est moins le cas dans les pays où l’accès aux soins est difficile, notamment sur le continent africain [22]. Ainsi, en cas de torsion testiculaire en Afrique, plus de 75 % des patients sont opérés au-delà de 24 heures d’évolution [27], et le taux de conservation n’est que de 58 % [28], ce qui en fait l’une des principales causes d’infertilité primaire du continent [3]. Plusieurs facteurs ont été évoqués pour expliquer ce retard de prise en charge diagnostique et thérapeutique: méconnaissance de la pathologie par les patients; accès limité, faute de moyens ou de disponibilité, aux établissements de santé; recours en premier lieu aux tradipraticiens ou aux pharmaciens [22, 23]. De plus, la méconnaissance de la pathologie par le corps médical peut également être à l’origine d’erreurs diagnostiques, estimées à plus de 50 % lors de consultations de soins primaires [27], et à l’origine d’un allongement majeur des délais de prise en charge [11], alors même que les espoirs de récupération diminuent grandement après 6 heures d’ischémie et sont nuls à 24 heures du début des symptômes [31].

La présentation clinique de la torsion du cordon spermatique en Afrique semble légèrement différer de celle des pays occidentaux: se manifestant également par une douleur testiculaire aiguë, avec une légère prédominance du côté gauche [7], son incidence moyenne semble plus faible, de 2,7 cas pour 100 000 hommes de moins de 25 ans par an, contre 1,4 en Asie, 3,8 en Amérique du Nord, ou 4,5 en Europe [2, 19]. Son pic de survenue est également plus tardif, vers 20-25 ans, alors qu’elle touche principalement les adolescents en Europe et aux États-Unis [23]. En outre, les études récentes menées sur le continent africain tendent à montrer une augmentation d’incidence à la deuxième et à la troisième décade: tout praticien doit donc garder en tête qu’elle peut toucher des hommes de tout âge [8]. Enfin, sa survenue semble favorisée, dans les pays tropicaux, par les températures plus fraîches et la baisse de l’humidité, en automne ou en hiver (période d’Harmattan) [13, 21].

De diagnostic aisé et basé sur l’examen clinique, et de traitement simple et peu consommateur de matériel, son traitement chirurgical est tout à fait réalisable en situation isolée. Pouvant être pratiquée par tout chirurgien quelle que soit sa spécialité, voire par un médecin généraliste formé aux principes chirurgicaux de base, cette intervention, réalisée dans des délais optimaux, est réalisable de façon ambulatoire et permet des suites opératoires simples. Dans la société africaine rurale, l’organisation communautaire place les soignants de première ligne au contact direct de la population: ceux-ci sont les principaux acteurs du système de soin, et dans ce contexte, la connaissance de cette pathologie et de son traitement est indispensable [3].

## Anatomie du testicule

Les testicules sont des organes pairs et asymétriques, localisés dans le scrotum. Cette position les fait bénéficier d’une température inférieure de quelques degrés à celle de l’abdomen, leur permettant d’assurer une double fonction, endocrine pour la stéroïdogenèse (synthèse de testostérone, à partir de la puberté) et exocrine pour la fonction reproductrice (spermatogenèse).

Organes mobiles, ils sont normalement retenus au sein de leurs enveloppes par trois points de fixations:

- le cordon testiculaire à leur extrémité supérieure;
- le mesorchium (ou fascia testiculaire) les fixant à la vaginale à leur face postérolatérale;
- le ligament testiculaire (reliquat du gubernaculum testis embryonnaire) à leur extrémité inférieure.

**Figure 1:**
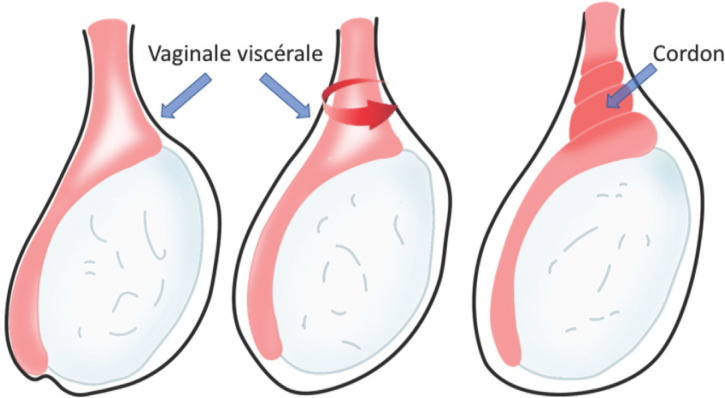
Déformation en « battant de cloche » dans la vaginale testiculaire, favorisant la rotation du testicule autour de son axe et la formation d’un ou plusieurs tours de spire sur le cordon spermatique. Collection Dr Chiron, HIA Bégin Bell-shaped deformity of the testicular vagina, favoring mobilization of the testicle around its axis and the formation of turns of the spermatic cord. Collection Dr Chiron, HIA Bégin

Ces moyens de fixations empêchent notamment les testicules de tourner autour de leur pédicule vasculaire. Au contraire, une fixation incomplète du testicule sur la tunique vaginale, notamment du fait de l’absence ou de l’insuffisance du mesorchium, entraîne une déformation dite « en battant de cloche » au sein de la vaginale: cette anomalie, où le testicule n’est fixé qu’à son pôle supérieur, est la plus souvent incriminée dans la genèse de la torsion testiculaire de l’adolescent ou du jeune adulte [4] (Fig. 1).

La vascularisation testiculaire est assurée par trois artères:

- l’artère spermatique, naissant directement de l’aorte à hauteur de L2-L3;
- l’artère déférentielle, branche de l’artère génito-vésicale issue de l’artère iliaque interne;
- l’artère funiculaire, branche de l’artère épigastrique inférieure issue de l’artère iliaque externe.

Ces trois artères s’anastomosent entre elles; toutefois, leur cheminement au sein du cordon spermatique rend cette vascularisation terminale, et toute torsion autour de l’axe vasculaire entraînera rapidement une ischémie et un risque de nécrose du testicule sous-jacent.

## Prise en charge

### Diagnostic clinique

La possibilité d’une torsion du cordon spermatique doit être évoquée dès la description des éléments cliniques.

À l’interrogatoire, le tableau de torsion du cordon spermatique est celui d’une douleur scrotale unilatérale, très intense, d’apparition brutale, chez un adolescent ou un jeune adulte. Elle peut parfois faire suite à un traumatisme testiculaire, ou à une activité sexuelle [27]. Cette douleur est rarement isolée, et peut s’accompagner classiquement de divers symptômes, au premier rang desquels les douleurs abdominales, ou des nausées et vomissements [23, 27]. Ceux-ci peuvent contribuer à l’errance diagnostique initiale, notamment chez l’enfant ou l’adolescent, et lorsqu’elle est présentée au premier plan, elle peut masquer dans presque 20 % des cas l’existence d’une torsion [29]. Pour cette population pédiatrique, certains auteurs ont pu récemment proposer un « score de risque » permettant de classer, d’après des éléments cliniques, le tableau clinique comme étant à risque faible, intermédiaire ou élevé de correspondre à une torsion testiculaire, qui peut aider à aiguiller vers ce diagnostic [24].

**Figure 2:**
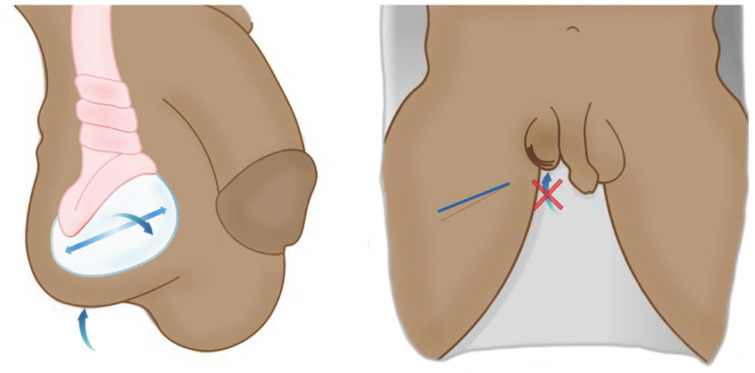
Signe de Gouverneur (ascension, horizontalisation, rétraction à l’anneau) et abolition du réflexe crémastérien (après stimulation de la face antéro-interne de la cuisse). Collection Dr Chiron, HIA Bégin Governor’s sign (ascension, horizontalization, retraction to the ring) and abolition of the cremasteric reflex (after stimulation of the antero-internal side of the thigh). Collection Dr Chiron, HIA Bégin

À l’examen clinique, du fait de la contraction réflexe du muscle crémaster, le testicule peut être ascensionné, horizontalisé, et rétracté à l’anneau inguinal à l’inspection (signe de Gouverneur). La palpation du testicule est rendue difficile voire impossible par l’intensité de la douleur et un testicule peu mobilisable. L’abolition du réflexe crémastérien, c’est-à-dire l’absence d’ascension du testicule lors de la stimulation du tiers supérieur et antéro-interne de la cuisse, est probablement le symptôme le plus spécifique (Fig. 2). Néanmoins, en cas de présentation tardive, ces signes cliniques typiques peuvent être pris en défaut [27].

En Afrique, plus de 50 % des tableaux cliniques de douleurs scrotales sont d’origines infectieuses [10]. Le diagnostic différentiel principal à écarter est l’orchiépididymite aiguë: dans ce cas, le patient présente également une « grosse bourse douloureuse aiguë », mais il s’y associe précocement fièvre et signes inflammatoires locaux. La notion de rapport sexuel à risque ou la présence de troubles urinaires concomitants peuvent étayer ce diagnostic. Le signe de Prehn (douleur soulagée par le soulèvement du testicule), typique des orchi-épididymites, est souvent négatif dans les torsions du cordon spermatique.

Dans tous les cas, il n’y a pas d’intérêt à réaliser des examens complémentaires, qui risquent de retarder inutilement la prise en charge chirurgicale, particulièrement en Afrique où les délais avant consultation sont souvent tardifs [1]. En particulier, malgré les progrès techniques de l’imagerie, l’apport de l’échographie reste faible: si elle permet, notamment par l’étude du flux Doppler, d’argumenter en faveur ou contre l’hypothèse d’une torsion testiculaire, elle reste un examen examinateur dépendant, qui ne sera pas toujours aisément disponible en soins primaires dans des délais très courts. De plus, on estime que le nombre d’explorations évitées par la réalisation de cet examen n’est que de 4 % [5]. Une échographie normale ne permet donc pas d’éliminer formellement une torsion.

La règle, dans ce contexte tout particulièrement, reste l’exploration chirurgicale dès lors que le diagnostic est suspecté et au moindre doute, situation fréquente à distance du début des symptômes [16]. Cette règle s’applique d’autant plus en population pédiatrique, où la douleur testiculaire peut être minime chez les jeunes enfants.

### Prise en charge thérapeutique

Pratiquée sans délai, l’objectif de la prise en charge est de rétablir en urgence la vascularisation du testicule, puis de le fixer afin de prévenir une nouvelle récidive. Au-delà de 12 heures après le début des symptômes, les chances de récupération sont faibles et l’orchidectomie est souvent nécessaire [15]. Il est important que le patient soit informé au préalable de ce risque.

### Détorsion manuelle

**Figure 3:**
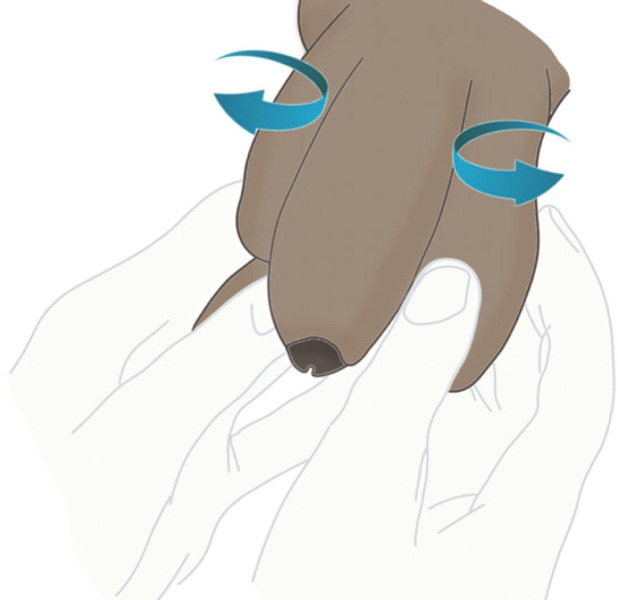
Détorsion manuelle externe. Elle vise à éloigner le pôle supérieur du testicule de la ligne médiane. Avec trois doigts on détord les cordons « comme un livre qu’on ouvre ». Collection Dr Chiron, HIA Bégin External manual untwisting. It is intended to move the upper pole of the testicle away from the median line. With three fingers the cords are untwisted “like opening a book”. Collection Dr Chiron, HIA Bégin

Le cordon testiculaire se tord habituellement de l’extérieur vers l’intérieur. La manœuvre de détorsion manuelle consiste à forcer la rotation du testicule dans le sens horaire à gauche et antihoraire à droite « comme un livre que l’on ouvre », en éloignant son pôle supérieur de la ligne médiane et en prenant comme repère les mains du soignant (Fig. 3).

La détorsion manuelle « doit être facile, ou ne pas être »: il ne faut donc pas insister en cas de résistance, ou si la douleur augmente (présence de plusieurs tours de spire) [20].

Elle n’a d’intérêt qu’à visée antalgique et pour gagner du temps avant l’accès au bloc opératoire lorsque l’on approche des 6 heures après le début des symptômes. Même en cas de sédation totale de la douleur, l’intervention chirurgicale reste alors indispensable: en effet, le cordon peut se tordre par plusieurs tours de spire et la détorsion manuelle pourrait être restée incomplète. Il faut donc sensibiliser les personnels soignants mais également les patients: la détorsion manuelle externe ne remplace pas la chirurgie et ne doit pas supprimer le sentiment d’urgence [9].

### Exploration testiculaire

Le patient est installé en décubitus dorsal, sous anesthésie générale ou rachianesthésie. L’antibioprophylaxie n’est pas nécessaire, de même que le sondage vésical. La verge peut être exclue du champ opératoire. Il sera nécessaire d’avoir une boîte de petite chirurgie, du fil tressé résorption lente 3/0 ou 2/0, du fil monobrin 4/0 ou 3/0 non résorbable, un bistouri électrique ou une pince bipolaire.

### Temps opératoires

#### Voie d’abord

**Figure 4:**
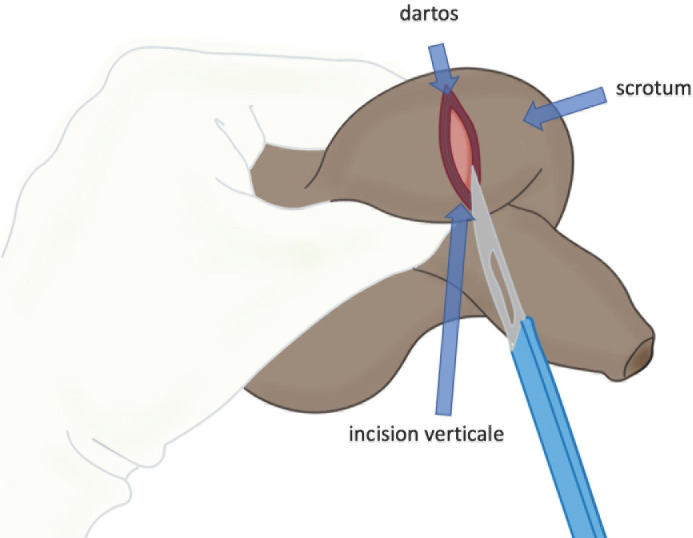
Incision horizontale du scrotum, sur le billot testiculaire mis en tension entre deux doigts Collection Dr Chiron, HIA Bégin Horizontal incision of the scrotum, using the testicle as a chopping block, tensioned by two fingers. Collection Dr Chiron, HIA Bégin

Si certaines écoles défendent l’incision verticale sur le raphé médian (permettant l’accès aux deux compartiments latéraux) [26], la scrototomie horizontale nous apparaît plus simple, plus rapide et suffisamment efficace pour le traitement de cette urgence, et donc mieux adaptée au contexte africain [16]. L’incision cutanée, réalisée à la lame froide et s’appuyant sur le billot testiculaire, est prolongée sur 3 à 4 cm (Fig. 4). Elle doit être suffisamment large pour permettre l’extériorisation atraumatique du testicule, mais également pour faciliter sa réintroduction intra-scrotale à la fin de la procédure.

#### Dissection-extériorisation

La dissection se poursuit à l’aide du bistouri électrique. Les différentes enveloppes sous-cutanées (muscle dartos, tunique celluleuse sous-cutanée, fascia spermatique externe et tunique fibreuse profonde) sont « discisées » aux ciseaux froids, isolées puis électrocoagulées, jusqu’à exposer la vaginale, qui n’est pas ouverte. Généralement, le testicule est extériorisé dans sa vaginale par l’incision cutanée.

#### Exploration-détorsion

Le diagnostic de torsion est évident quand les tours de spire sont visibles sur le cordon extra-vaginal. La détorsion se fait alors manuellement et sans difficulté, par manœuvre de rotation du cordon jusqu’à ce qu’il n’y ait plus de tour de spire visible. L’absence de tour de spire sur le cordon n’exclut pas le diagnostic, car la détorsion peut avoir lieu à l’induction de l’anesthésie, par le relâchement musculaire du muscle crémaster qu’elle induit [17].

**Figure 5:**
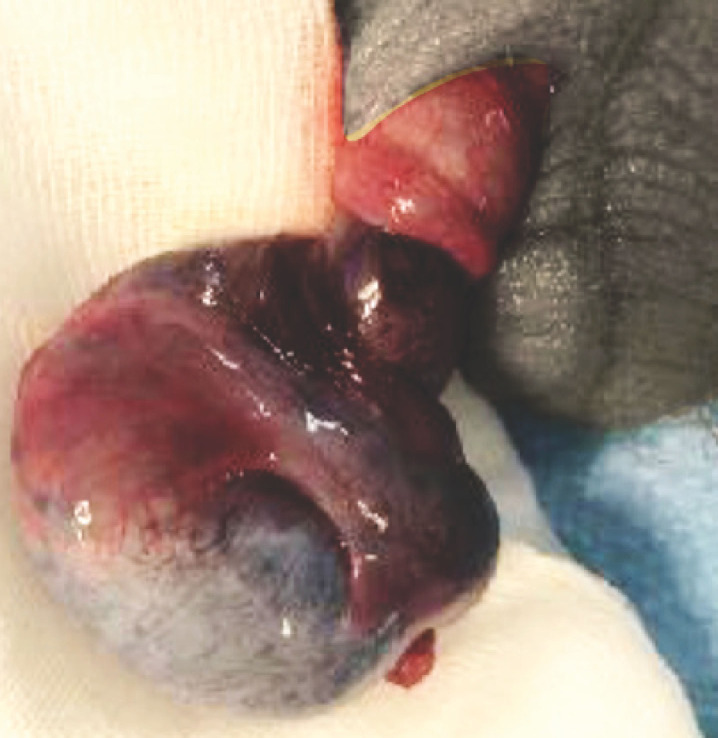
Torsion du cordon spermatique, avec double tour de spire et ischémie testiculaire. Collection Pr Savoie, HIA Sainte-Anne Twisting of the spermatic cord, with double turn and testicular ischemia. Collection Pr Savoie, HIA Sainte-Anne

Dans tous les cas, l’ouverture de la tunique vaginale est impérative: d’une part, la torsion peut être intravaginale (fréquente chez l’enfant, mais rare chez l’adulte) et cela permet de ne pas la méconnaître; d’autre part, seule cette ouverture permettra d’estimer l’aspect du testicule. La couleur de la pulpe testiculaire, visualisée à travers l’albuginée, témoigne du niveau de souffrance ischémique: elle peut être rosée, violacée ou noire (Fig. 5). En l’absence de traumatisme, le diagnostic peut également être porté devant un aspect bleuté de la pulpe.

**Figure 6:**
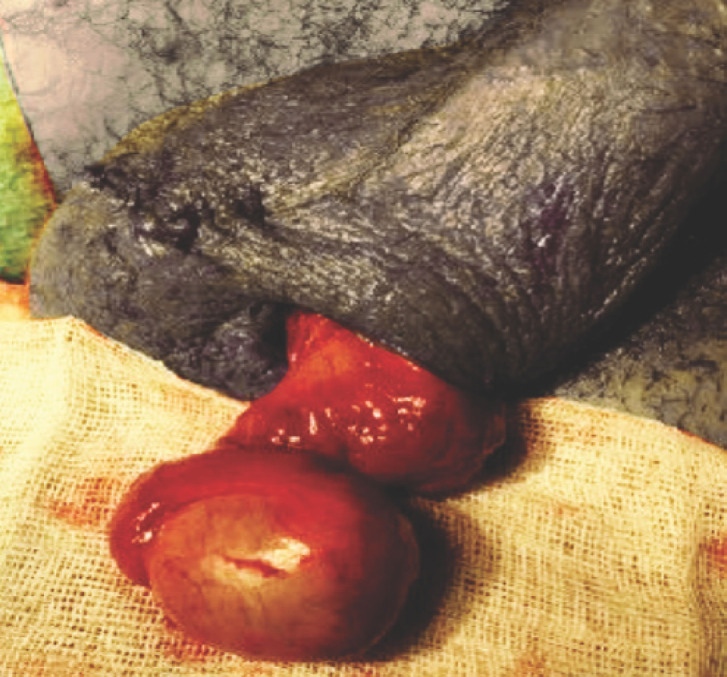
Après détorsion, recoloration du testicule au bout de quelques minutes. Collection Pr Savoie, HIA Saint-Anne After untwisting, recoloring of the testicle after a few minutes. Collection Pr Savoie, HIA Saint-Anne. Collection Pr Savoie, HIA Sainte-Anne

Après détorsion, deux situations sont possibles. Soit le testicule reprend, spontanément et en quelques minutes, une couleur rosée (Fig. 6): il est viable et peut être conservé.

Soit le testicule ne se recolore pas: dans ce cas, il peut être trempé dans du sérum physiologique chaud pendant 5 minutes afin de provoquer une vasodilatation [30]. Des injections de vasodilatateur (lidocaïne non adrénalinée, papavérine ou vérapamil) dans le cordon spermatique ont été proposées [25].

Les testicules restant noirs ou hémorragiques au-delà de ces 5 minutes peuvent être considérés comme non fonctionnels, et une orchidectomie est recommandée [15]. En Afrique, les retards de prise en charge conduisent souvent à des ischémies plus avancées, et plus de 20 % des patients se présentent d’emblée avec un tableau de gangrène des testicules [18]. Dans ce cas, l’orchidectomie est inévitable afin d’éviter les complications septiques d’une torsion dépassée.

Il faut alors ligaturer le cordon au sommet de la vaginale, par un point appuyé de fil résorbable tressé de décimale 0. Le testicule ne reste alors fixé qu’à son pôle inférieur par le gubernaculum testis, qui est sectionné entre deux ligatures au plus près du testicule. La pièce opératoire devra ensuite être placée dans du liquide fixateur, en vue d’une étude anatomopathologique.

#### Prévention de la récidive par orchidopexie

Si le testicule est viable et qu’il est conservé, il est indispensable de le fixer afin de prévenir la récidive. La technique de choix de l’orchidopexie consiste en une fixation par triangulation, en trois points, avec éversion, suture et fixation de l’albuginée et de la vaginale au muscle dartos.

**Figure 7:**
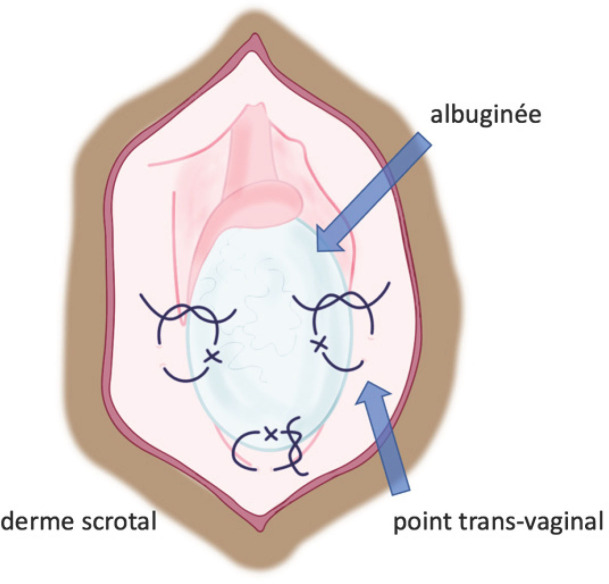
Fixation transvaginale par deux points latéraux et un point au pôle inférieur. Collection Dr Chiron, HIA Bégin Transvaginal fixation by two lateral points and one point at the lower pole. Collection Dr Chiron, HIA Bégin

**Figure 8:**
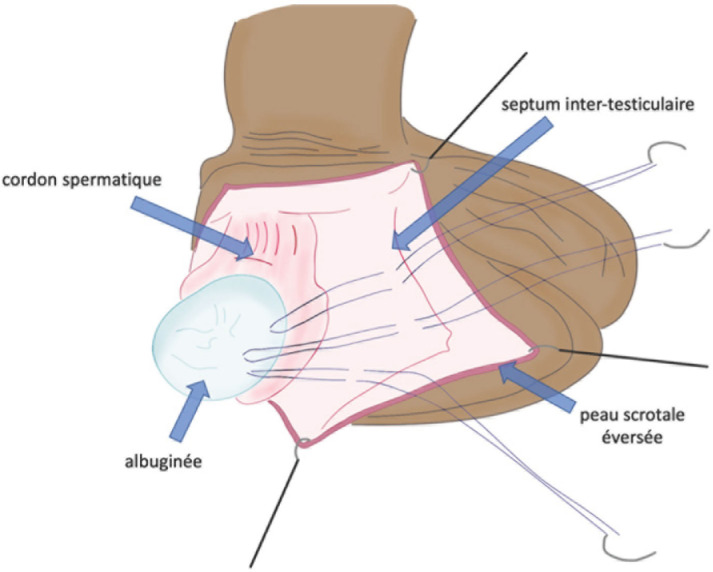
Méthode de fixation testiculaire par trois points séparés entre l’albuginée et le septum médian. Collection Dr Chiron, HIA Bégin Testicular fixation method with three separate points between the albuginea and the median septum. Collection Dr Chiron, HIA Bégin

Ces trois points de fixation, disposés latéralement de part et d’autre du testicule et à son pôle inférieur, et utilisant un fil monobrin non résorbable 3/0 ou 4/0, rendent impossible toute nouvelle rotation du testicule dans le scrotum (Fig. 7). Une précaution particulière doit être prise lors du passage des points à travers l’albuginée, en restant le plus superficiel possible (passage interne tangentiel à l’albuginée) pour éviter au maximum la pulpe testiculaire sous-jacente. Les points traversent ensuite la vaginale pour s’appuyer sur le dartos: pour cela, il faut éverser l’ensemble de la paroi scrotale avec un doigt ou un instrument. Une fois réalisés, il est nécessaire de s’assurer que les points de suture pariétaux ne transfixient pas la peau. Alternativement, certains auteurs proposent de réaliser tous les points de fixation à la face interne du testicule, appuyés sur le septum médian du scrotum, afin d’éviter la possibilité de palpation des points à travers la peau, parfois gênante pour les patients (Fig. 8).

Si la torsion du cordon spermatique est constatée ou suspectée sur les données peropératoires, l’orchidopexie est conseillée. Au contraire, en cas de diagnostic différentiel évident (épididymite, traumatisme), elle n’est pas indispensable et même déconseillée.

#### Orchidopexie controlatérale

Il est fréquent que les deux testicules soient atteints par la même anomalie anatomique favorisant la torsion. Une intervention à visée préventive du côté opposé doit alors être proposée. La nécessité ou non de réaliser cette orchidopexie controlatérale dans le même temps fait débat (absence de bénéfice immédiat, augmentation du risque de complication post-opératoire…) [12], et une revue systématique de la littérature est à l’étude pour définir un consensus robuste sur ce point [6]. Toutefois en Afrique, la balance bénéfices/risques prenant notamment en compte la difficulté d’accès ultérieurs aux soins justifie, de notre point de vue, la réalisation d’une orchidopexie controlatérale dans le même temps. L’abord controlatéral nécessitera alors une deuxième incision transversale en regard du second testicule.

#### Fermeture

En cas d’orchidopexie simple, la fermeture se fait sans drainage. En cas d’orchidectomie, un drainage est indiqué dans un contexte de sepsis ou de fonte purulente du testicule, par exemple à l’aide d’une lame déclive extériorisée par une contre-incision inférieure.

La vaginale n’est pas obligatoirement refermée. Le dartos est refermé à l’aide d’un surjet ou de points séparés en utilisant un fil tressé à résorption lente 2/0 ou 3/0. Pour améliorer l’hémostase, ce surjet peut être « passé ». La peau scrotale est refermée à l’aide de points séparés de fil monobrin à résorption rapide 3/0, permettant de limiter les soins infirmiers en post-opératoire. La cicatrice est recouverte par un pansement sec quelques jours, puis sera laissée à l’air. L’utilisation d’un pansement compressif est possible.

#### Complications post-opératoires

Les principales complications sont l’hématome et l’infection. Il faut garder à l’esprit que l’observance quant au suivi post-opératoire est rare dans les pays en développement, notamment lorsque les suites post-opératoires sont simples. Une gestion ambulatoire est souvent la règle.

## Conclusion

La prise en charge de la torsion testiculaire se prête idéalement à la chirurgie en situation isolée, comme c’est souvent le cas en Afrique. Le tableau clinique est suffisant pour le diagnostic et la prise de décision thérapeutique. La chirurgie est indispensable, même en cas de possible rajout manœuvre de détorsion manuelle efficace. À la portée de tout chirurgien, le geste est simple, peu coûteux en matériel, personnel et soins post-opératoires. L’anatomie en plusieurs tuniques du testicule facilite sa fixation chirurgicale en cas de conservation, par des points idéalement en triangulation. En fonction de l’aspect du testicule et, dans une moindre mesure, de la durée d’évolution des symptômes, l’orchidectomie peut être nécessaire. L’apprentissage de la gestion d’une torsion du cordon spermatique par le personnel médical et chirurgical amené à intervenir en situation isolée en Afrique permettrait d’améliorer les délais de prise en charge et de réduire le taux d’orchidectomie.

## Liens d’intérêts

Les auteurs ne déclarent aucun lien d’intérêt.

## Contribution des auteurs

T. Long-Depaquit: Rédaction du manuscrit

P. Chiron: Réalisation des illustrations

S. Bourgouin: Relecture du manuscrit

J. Hardy: Relecture du manuscrit et traduction abstract en anglais

F.-X. Deledalle: Relecture du manuscrit

J. Laroche: Relecture du manuscrit et bibliographie

B. Molimard: Relecture du manuscrit

P.-H. Savoie: Coordination de la rédaction du manuscrit, relecture et correction du manuscrit.
